# Neurons with dendrites can perform linearly separable computations with low resolution synaptic weights

**DOI:** 10.12688/f1000research.26486.3

**Published:** 2021-04-18

**Authors:** Romain D. Cazé, Marcel Stimberg

**Affiliations:** 1 IEMN, CNRS UMR 8520, Villeneuve d'asq, 59650, France; 2 Institut de la vision, CNRS, INSERM, Paris, 75012, France

**Keywords:** Dendrites, computation, linearly separable, implementation

## Abstract

In theory, neurons modelled as single layer perceptrons can implement all linearly separable computations. In practice, however, these computations may require arbitrarily precise synaptic weights. This is a strong constraint since both biological neurons and their artificial counterparts have to cope with limited precision. Here, we explore how non-linear processing in dendrites helps overcome this constraint. We start by finding a class of computations which requires increasing precision with the number of inputs in a perceptron and show that it can be implemented without this constraint in a neuron with sub-linear dendritic subunits. Then, we complement this analytical study by a simulation of a biophysical neuron model with two passive dendrites and a soma, and show that it can implement this computation. This work demonstrates a new role of dendrites in neural computation: by distributing the computation across independent subunits, the same computation can be performed more efficiently with less precise tuning of the synaptic weights. This work not only offers new insight into the importance of dendrites for biological neurons, but also paves the way for new, more efficient architectures of artificial neuromorphic chips.

## Introduction

In theoretical studies, scientists typically represent neurons as linear threshold units (LTU; summing up the weighted inputs and comparing the sum to a threshold) ^1^. Multiple decades ago, theoreticians exactly delimited the computational capacities of LTUs, also known as perceptrons ^2^. LTUs cannot implement computations like the exclusive or (XOR), but they can implement all possible linearly separable computations and a sufficiently large network of LTUs can approximate all possible computations ^3^.

Research in computer science investigated the synaptic weight resolution required to implement linearly separable computations ^4, 5^. Hastad *et al*. studied a computation implementable by an LTU only if its synaptic weight resolution grows exponentially with the number of inputs. We consider, similarly to these studies, the needed resources as the minimal size of integer-valued weights necessary to implement a set of linearly separable computations.

Requiring a high synaptic resolution has important consequences. In the nervous system, neurons would need to maintain a large number of synapses or synapses with a large number of stable states. For the same reason, neuromorphic chips based on LTUs have to dedicate a large amount of resources to synapses ^6^. We demonstrate here that dendrites might be a way to cope with this challenge.

Dendrites are the receptive elements of neurons where most of the synapses lie. They turn neurons into a multilayer network ^7, 8^ because of their non-linear properties ^9, 10^. These non-linearities enable neurons to perform linearly inseparable computations like the XOR or the feature binding problem ^11, 12^. The non-linear integration also appears to be tuned for efficient integration of *in vivo* presynaptic activity ^13^.

In this study, we investigate whether dendrites can also decrease the synaptic resolution necessary to implement linearly separable computations. We address this question by looking at all the computations of three input variables implementable by an LTU with positive synaptic weights. We then extend the definition of one of these computations to an arbitrarily high number of inputs. Finally, we implement this computation in a biophysical neuron model with two passive dendrites using fewer synapses than an LTU.

This work proposes a new role for dendrites in the nervous system, but also paves the way for a new generation of more cost-efficient artificial neural networks and neuromorphic chips composed of neurons with dendrites.

## Methods

### Biophysical neuron model

We performed simulations in a spatially extended neuron model, consisting of a spherical soma (diameter 10 µm) and two cylindrical dendrites (length 400 µm and diameter 0.4 µm). The two dendrites are each divided into four compartments and connect to the soma at one extremity.

In contrast to a point-neuron model, each compartment has a distinct membrane potential.

The membrane potential dynamics of the somatic compartment follows the Hodgkin-Huxley formalism with:

$$C_{m}\frac{dV_{\text{soma}}}{dt}=g_{L}(V-E_{L})+{\bar{g}}_{K}n^{4}(V-E_{K})+{\bar{g}}_{Na}\;m^{3}h(V-E_{Na})+I_{a}+I_{s}\;(1)$$

The dendritic compartments only contain passive currents:

$$C_{m}\frac{dV_{\text{dend}}}{dt}=g_{L}(V-E_{L})+I_{a}+I_{s}\;(2)$$

Here, *V*_soma _and *V*_dend _are the respective membrane potentials, *C _m_* = 1µFcm ^−2^ is the membrane capacitance, *g _L_*, $\bar{g}$ *_K_*, and $\bar{g}$ *_Na_* stand for the leak, the maximum potassium and sodium conductances respectively, and *E _L_*, *E _K_*, and *E _Na_* stand for the corresponding reversal potentials. The currents *I _a_* represent the axial currents due to the membrane potential difference between connected compartments. The synaptic current *I _s_* arises from a synapse placed at the respective compartment. It is described by

$$I_{s}=g_{s}(E_{s}-V)\;(3)$$

with *E _s_* being the synaptic reversal potential and *g _s_* the synaptic conductance. This conductance jumps up instantaneously for each incoming spike and decays exponentially with time constant *τ _s_* = 1 ms otherwise:

$$\frac{dg_{s}}{dt}=-\frac{g_{s}}{\tau_{s}}\;(4)$$

The dynamics of the gating variables *n*, *m*, and *h* are identical to 14, except for shifting the membrane potential relative to *V _T_* = –50 mV instead of the cell’s resting potential. The equations are omitted here for brevity. The parameter values are summarized in Table 1. Note that due to the absence of sodium and potassium channels in the dendrites, the dendrites are passive and cannot generate action potentials.

**Table 1.** Parameter values used in the biophysical model.

Equilibrium potentials (in mV)		Conductances (in mS/cm ^2^)	
*E _L_*	−65	*g _L_*	10
*E _Na_*	50	$\bar{g}$ *_Na_*	100
*E _K_*	−90	$\bar{g}$ *_K_*	30
*E _s_*	0		

All simulations were performed with Brian 2 ^15^. The code is available at http://doi.org/10.5281/zenodo.4315011 ^16^. It allows for reproducing the results presented in Figure 4, Figure 5 and Figure 6. To demonstrate that the details of the neuron model do not matter for the results presented here, the provided code can also be run with a simpler leaky integrate-and-fire model.

### Elementary neuron model and Boolean functions

As a reminder, we first define Boolean functions:

**Definition 1.** *A Boolean function of n variables is a function on* {0, 1} ^*n*^ *into* {0, 1}, *where n is a positive integer.*

Note that we use the terms *function* and *computation* interchangeably.

A special class of Boolean functions, which are of particular relevance for neurons, are linearly separable computations:

**Definition 2.** *f is a linearly separable computation of n variables if and only if there exists at least one vector w* ∈ ℝ *^n^* *and a threshold* Θ ∈ ℝ *such that:*

$$f(X)=\{\begin{array}{cc} 1 & if\;w\cdot X\geq \Theta \\ 0 & otherwise \end{array}$$

*where X* ∈ {0, 1} *^n^ is the vector notation for the Boolean input variables.*

Binary neurons are one of the simplest possible neuron models and closely related to the functions described above: their inputs are binary variables, representing the activity of their input pathways, and their output is a single binary variable, representing whether the neuron is active or not. The standard model is a linear threshold unit (LTU), defined as follows:

**Definition 3.** *An LTU has a set of m weights* *w _i_* ∈ *and a threshold* Θ ∈ *so that:*

$$f(X)=\{\begin{array}{cc} 1 & if\;\sum_{i=1}^{m}w_{i}X_{i}\geq \Theta \\ 0 & otherwise \end{array}$$

*where X* = ( *X*_1_, . . . , *X _m_*) *are the binary inputs to the neuron, and and are the possible values for synaptic weights and the threshold, respectively.*

This definition is virtually identical to Definition 2, however, *w _i_* and Θ are no longer arbitrary real values, but chosen from a finite set of numbers depending on the specific implementation and noise at which these value can be stabilised. It follows that a neuron may not be able to implement all linearly separable functions. For instance, a neuron with non-negative weights can only perform positive linearly separable computations:

**Definition 4.** *A threshold function f is positive if and only if* *f* ( *X*) ≥ *f* ( *Z*) ∀( *X* , *Z*) ∈ {0, 1} *^n^* *such that X* ≥ *Z* ( *meaning that* ∀ *i: x _i_* ≥ *z _i_*).

To account for saturation occurring in dendrites, we introduce the sub-linear threshold unit (SLTU):

**Definition 5.** *An SLTU with d dendrites and n inputs has a set of d* × *n weights w _i_*_, *j*_ ∈ {0, 1} *with n w _i _such that* $\sum_{j=1}^{d}w_{i,j}\;=w_{i}\;$ *and a threshold* Θ ∈ , *such that:*

$$f(X)=\{\begin{array}{cc} 1 & if\;\sum_{i=1}^{d}E\left(\sum_{j=1}^{n}w_{i,j}X_{i,j}\right)\geq \Theta \\ 0 & \mathrm{otherwise} \end{array}$$

*with*

$$E(Y)=\{\begin{array}{cc} 1 & if\;Y\geq 1\;\\ Y & otherwise \end{array}$$

The function *E* accounts for dendritic saturation; because we work with binary weights its value is either 0 or 1.

Such a neuron model can implement all positive Boolean computations (see Definition 4) given a sufficient number of dendrites and synapses ^11^.

We used integer-valued and non-negative parameters both for the LTU and the SLTU without loss of generality. It allows us to exactly determine the minimal resources necessary to implement a given computation.

## Results

### Implementation of computations with three input variables

**Figure 1.**
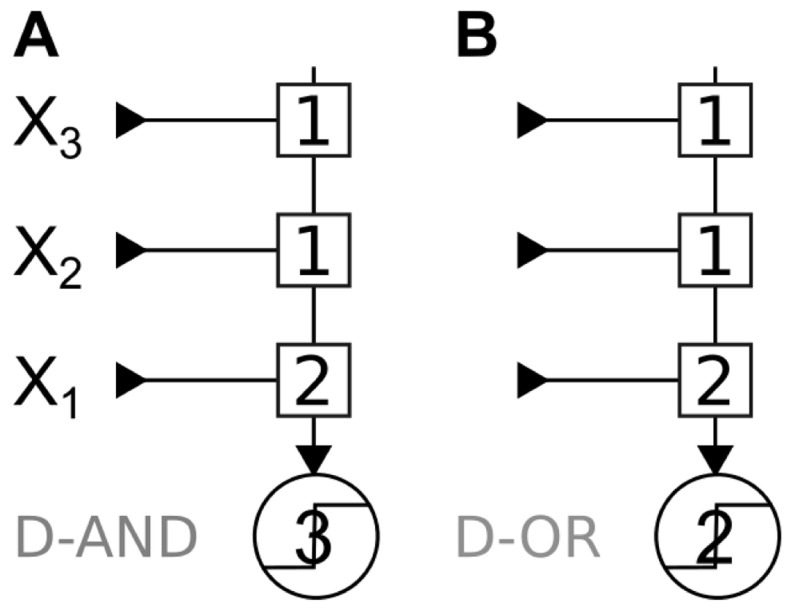
Minimal implementation of the Dominant AND computation (D-AND) and its dual by a linear threshold unit (LTU). Implementations of the D-AND where *X*_1 _is the dominant input. Squares represent synapses with their synaptic weight, and circles stand for transfer functions. Here, the transfer functions are threshold functions with the given value as their threshold. **A**: Implementation of the D-AND, note that *X*_1 _has twice the synaptic weight compared to the others. **B**: Implementation of the D-OR, note that we keep the same synaptic architecture and we only change the threshold of the transfer function.

We begin by listing all computations of *n* = 3 inputs that are implementable by an LTU (i.e., positive threshold functions; Table 2). These computations can be divided in five classes, and one can obtain all computations from a class by swapping the input labels. The OR, AND/OR, and AND can be implemented with equal synaptic weights. In contrast, the remaining classes require heterogeneous synaptic weights. We call these classes the Dominant AND (D-AND) and the Dominant OR (D-OR): to implement these computations, an LTU needs to have one synaptic weight that is twice as big as the others (see Figure 1). 

**Table 2.** The five classes of positive threshold functions for *n* = 3 inputs with their associated truth tables. We have assigned a name to each class for easier reference.

Inputs	OR	AND/OR	AND	D-OR	D-AND
000	0	0	0	0	0
001	1	0	0	0	0
010	1	0	0	0	0
011	1	1	0	1	0
100	1	0	0	1	0
101	1	1	0	1	1
110	1	1	0	1	1
111	1	1	1	1	1

The D-AND computation gets its name from the fact that it requires the activation of a dominant (D) input AND the activation of another input. The D-OR is the Boolean dual of the D-AND, i.e. obtained by replacing AND operations by OR, and vice versa. In this computation, activation of the dominant input OR of the two other inputs together triggers an output. Both computations have a “dominant input” – an input that is *sufficient* to make the output true (D-OR), respectively *necessary* to make the output true (D-AND). There is nothing comparable in the other three computations, which treat all inputs identically. In the present paper, we always chose *X*_1 _as the dominant input, but we could have picked *X*_2_ or *X*_3_. 

An LTU ( Figure 1) implements D-AND and D-OR by making use of synaptic strength to distinguish between the dominant and non-dominant inputs. We employed synaptic weights with integer values to reflect their finite precision. Even if synaptic weights can take real values, a finite precision means a finite number of values, which again can be represented by an integer value. The weight and threshold values to implement a function are obviously not unique. For example, we could multiply all the weights by 2 and set the threshold to 6 (D-AND), or 4 (D-OR) and obtain the same results. Here, we always use the lowest possible integer values for synaptic weights, and the corresponding lowest possible threshold.

Next, we wanted to implement the D-AND and D-OR computation in threshold units with non-linear dendritic sub-units, as an abstraction of neurons with dendrites ^7^.

We consider two types of non-linearities: a threshold function to model supra-linear summation; and a saturating function to model sub-linear summation (SLTU; see Methods). Both types of summation have been observed in dendrites. Dendritic spikes are a well-known example of supra-linear summation ^12^, while sub-linear summation can be observed in completely passive dendrites due to a reduced driving force ^9^.

**Figure 2.**
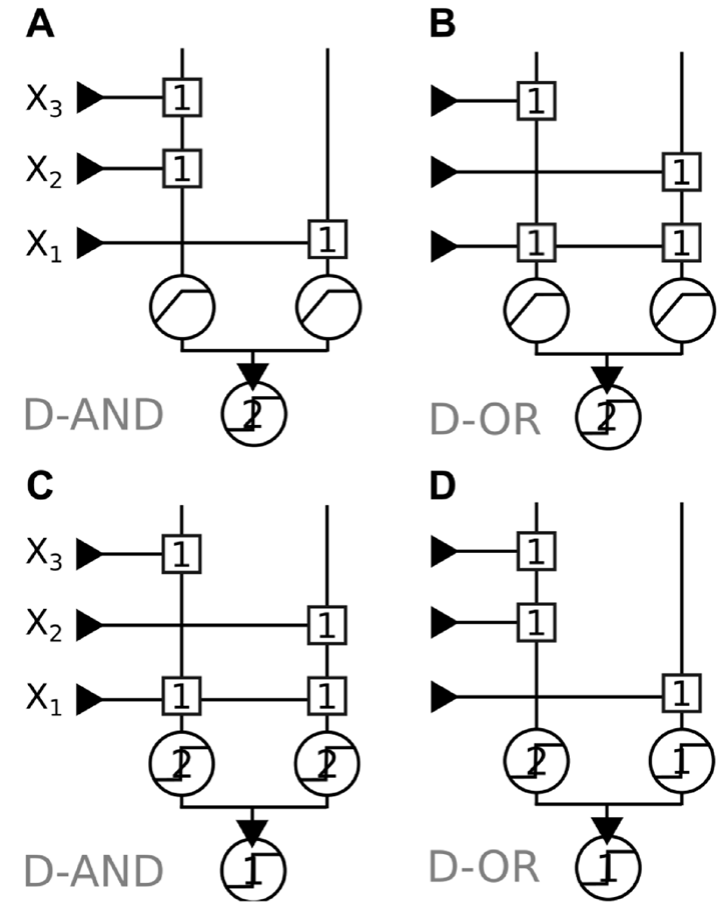
Minimal implementation of the Dominant AND computation (D-AND) and its dual (D-OR) by a neuron with dendrites. Squares represent synapses and circles represent transfer functions with their respective threshold/saturation values. Note that the final transfer functions (“somatic integration”) are always threshold units, whereas the transfer functions of the sub-units (“dendrites”) are threshold functions for supra-linear summation, and saturating functions (corresponding to the *E* function defined in Definition 5) for strictly sub-linear summation. **A**: D-AND implementation using sub-linear summation where *X*_1 _targets only one dendrite. **B**: D-OR implementation, in this case *X*_1 _targets two sub-linear dendrites. **C**: D-AND implementation using supra-linear summation, where *X*_1 _targets two dendrites. **D**: D-OR implementation, *X*_1_ in this case targets only one dendrite.

On the one hand, Figure 2 (top) shows that a neuron with supra-linear dendrites implements the D-OR using space whereas the sub-linear implementation uses strength. On the other hand, Figure 2 (bottom) shows that a neuron with supra-linear dendrites implements the D-AND using strength whereas the sub-linear implementation uses space.

In both cases, all synapses are of identical strength. However, note that in the supra-linear implementation of the D-AND in Figure 2C the *X*_1_ input connects to both dendrites. Therefore, if we define an input’s synaptic weight as the total effect it has in the final summation stage (analogous to depolarisation measured in the soma of a neuron), we have to consider the weight of *X*_1 _as twice as high as the other inputs. This makes this implementation “as bad as” the implementation in an LTU ( Figure 1A): the dominance of *X*_1 _is expressed by a stronger weight.

This starkly contrasts with the sub-linear implementation of the D-AND ( Figure 2D), where all synaptic weights are identical. The placement of *X*_1_’s synapse causes its dominance: while *X*_2 _and *X*_3 _share a dendrite, *X*_1_’s synapse lies alone on a dendrite. This implementation uses space. We focus on sub-linear summation and the D-AND for the rest of the study.

### Implementing the D-AND for an arbitrary number of input variables

In the previous section, we have limited our analysis to computations with three input variables. We will now extend the definition of the D-AND to an arbitrary number of input variables. As in the three-variables case, we will consider one input to be the dominant input (assumed to be *X*_1_, without loss of generality). This input has to be activated together with at least one of the non-dominant inputs. Formally, we therefore define *f _n_*( *X*) as follows:

$$f_{n}(X)=X_{1}\wedge \left(\overset{n}{\underset{i=2}{V}}X_{i})\;(5\right)$$

where *X* is the *n*-dimensional input vector with elements *X*_1_... *X _n_*.

**Figure 3.**
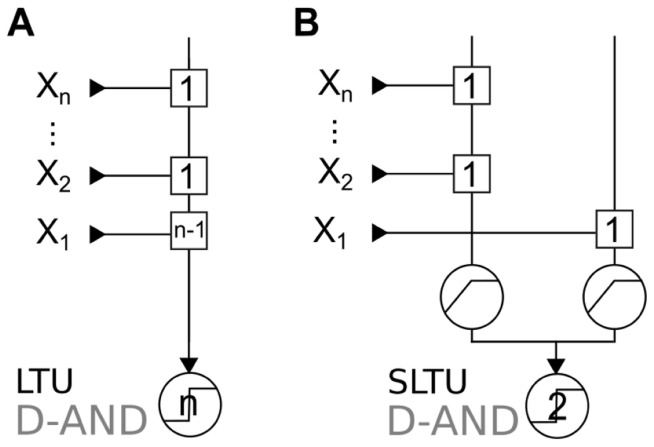
Extending the D-AND implementation to *n* inputs. Synaptic weights are in squares, and transfer functions are in circles. **A**: Minimal D-AND implementation in an LTU. Note that this implementation requires a synaptic weight that is *n* − 1 times bigger than the smallest weight. **B**: Implementation in an SLTU with sub-linear summation (see Definition 5).

We can implement this computation in an LTU ( Figure 3A), as well as in an SLTU ( Figure 3B)

In the LTU implementation ( Figure 3A), the D-AND of *n* variables requires that an input has a synaptic weight at least *n* − 1 times bigger than the other inputs, and the threshold has to grow accordingly.

We can summarise these observations in a proposition.

**Proposition 1.** *To implement the D-AND, an LTU requires that an input has a synaptic weight n* − 1 *times bigger than the smallest synaptic weight.*

*Proof.* The LTU must stay silent when *X*_1_ is not active, even if *X*_2_, *X*_3_, . . . , *X _n_* are active. Therefore *w*_2_ + *w*_3_ + ... + *w _n_* < Θ, thus Θ must be at least *n* × *w _min_* with *w _min_* the smallest synaptic weight.

Conversely, the output should be active as soon as *X*_1_ is co-active with any other input *X _j _* (for *j* > 1). So *w*_1_ + *w _min_* ≥ Θ, this means *w*_1_ + *w _min_* ≥ *n* × *w _min_*, thus *w*_1_ ≥ *w _min_*( *n* − 1).

In contrast, Figure 3B provides a constructive proof that an SLTU can implement the D-AND with equal synaptic weights. In this implementation, the distinguishing feature of the dominant input is that it targets the second dendrite; synaptic weights and the threshold do not have to change with the number of inputs. If one only measured the response to single inputs at the “soma” (last stage of summation), the dominant input would be indistinguishable from the other inputs, despite its dramatically different importance.

We will see next how these insights transfer to a more realistic biophysical model.

### Implementation of the D-AND in a biophysical model

**Figure 4.**
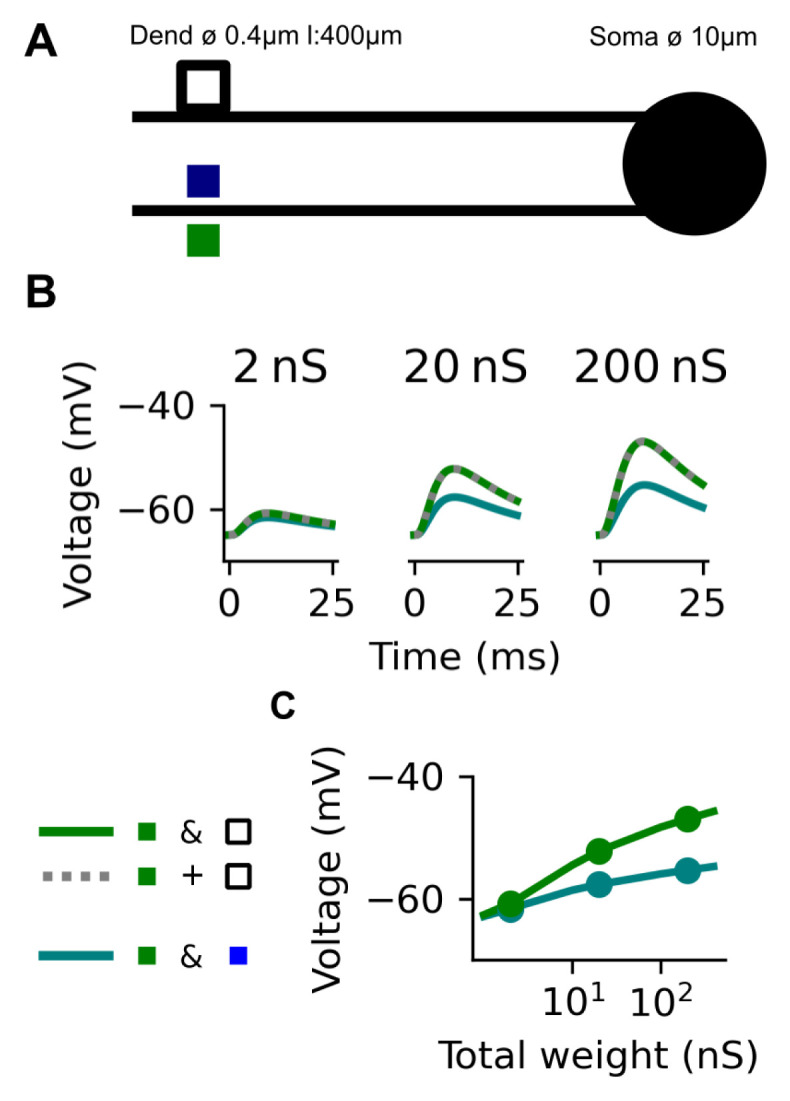
A biophysical model sensitive to synapses’ spatial distribution. **A**: A biophysical model with two dendrites and a soma (lines: dendrites, circle: soma). Coloured squares depict synapses. The model has three equivalent groups of synapses (black edges/blue/green). **B**: Somatic membrane voltage traced in 3 scenarios: either two groups of synapses activated simultaneously (& symbol) or we linearly added the response from two synaptic groups (+ symbol) note that the green and dotted grey line overlay **C**: Maximal membrane voltage at the soma depending on the total synaptic weight for either clustered (aquamarine) or dispersed (green) stimulation. We omitted the grey dotted line here as it overlays with the green.

Figure 4A presents a biophysical model of a single neuron implementing the D-AND computation with three groups of synapses. All the synapses, taken individually, produced the exact same depolarisation at the soma because we place them at the same distance (350 µm) and give them the same maximal conductance (20 nS).

We first look at the sub-threshold behaviour by disabling the sodium channels in the soma ( $g_{Na}^{max}$ = 0). Figure 4B plots the somatic voltage response at distinct locations in response to either clustered (black) or dispersed (aquamarine) synaptic activation. Despite activating the same number of synapses in both cases, and despite them all having the same strength, the depolarisation is markedly different. When we disperse active synapses, EPSPs sum linearly (same as dotted gray line) whereas when we cluster active synapses summation becomes sub-linear. This difference is robust with respect to the specific values of the synaptic weights. As shown in Figure 4C, the dispersed activation always exceeds the clustered activation, for the same total synaptic weight. This difference remains even for a total weight bigger for the clustered than the dispersed case. For example, a clustered activation with a total weight of 100 nS leads to a maximum membrane potential of only −54mV in the soma, whereas a dispersed activation with a mere total weight of 10 nS leads to a maximal membrane potential of −52.5mV.

We can explain this observation by considering the synaptic driving force ^17^. The synaptic current induced by the activation of the synapse depends on the distance between the membrane potential and the synapses’ reversal potential; when several inputs drive the membrane potential closer to the reversal potential (here 0mV), this driving force diminishes. The combined effect of multiple synaptic inputs is therefore smaller than what is expected from summing the individual effects. In other words, the dendrite performs sub-linear summation.

This means that even if we have a complete synaptic democracy ^18^ (all synapses have the same impact on the soma when taken individually), the relative placement of the synapses strongly influences the somatic response.

Based on the sub-threshold behaviour presented above, we will now show that we can implement the D-AND in a spiking neuron model. It is crucial to look at the supra-threshold behaviour as it is how the neuron communicates with the rest of the network. Moreover, backpropagated action potentials might undermine the dendritic non-linearity disrupting the implementation ^19^.

**Figure 5.**
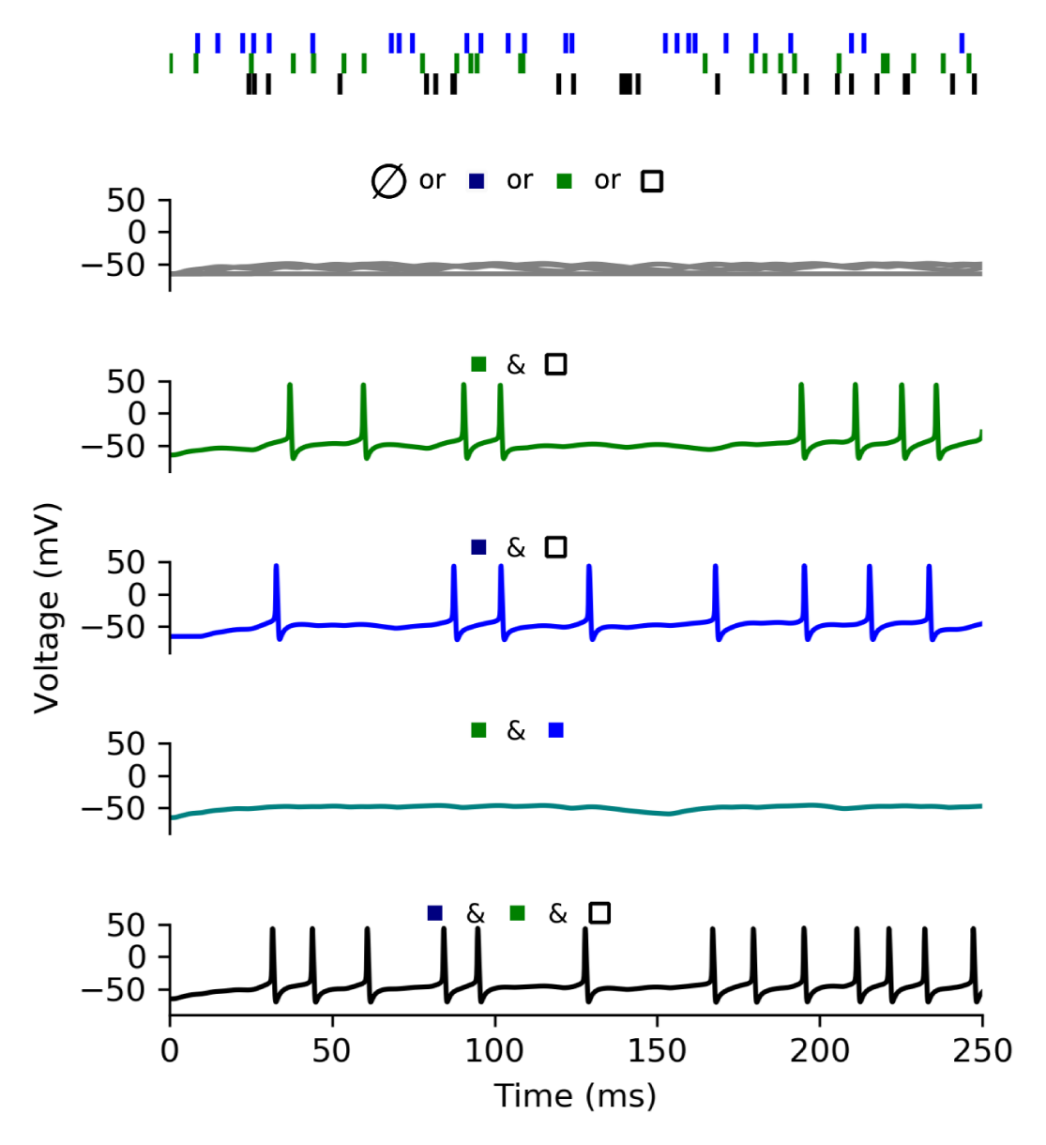
A biophysical model implementing the Dominant AND (rate interpretation). We show in this figure how the model presented in the previous figure responds in 8 different cases. *X _i_* = 1 corresponds to a presynaptic neuron firing at 100Hz and the 8 cases correspond to the truth table. Top: activity of the three input synapses, the two first synapses impinge on the same dendrite while the black one impinges on another. Bottom: Eight somatic membrane responses depending on the active inputs. (gray: no synapse/only black/green/blue, green: black + green, blue: black + blue, aquamarine: green + blue, black: all inputs active). The difference between the aquamarine line (green and blue inputs) and the green and blue lines (black input and either green or blue input) is due to the sub-linear summation in the dendrite. With linear summation these three responses would have been identical -either all firing or not.

We can interpret Boolean inputs and outputs in different ways when we apply them to a biophysical spiking neuron model. Here, we will consider two interpretations. Firstly, we can think of an active input as corresponding to a continuous stimulation where the individual spikes arrive at random times, and of an active output as some spiking activity of the neuron (“rate interpretation”). Alternatively, we can think of active inputs as coincidentally arriving spikes within a certain time window, and accordingly of an active output as a single spike emitted in response (“spike interpretation”). We present the model implementing the rate interpretation in Figure 5. We introduced this model earlier ( Figure 4), except that it now has active sodium channels in the soma ( $g_{Na}^{max}$ = 650mS cm ^–2^). Each of its inputs (colours corresponding to the colours in Figure 4) activates in 25 randomly chosen time-bins of 1 ms to simulate a 100 Hz spike train over 250 ms.

The Figure 5 displays, from top to bottom, the model’s responses in five different situations:

- A single input activates, in this case the neuron remains silent. We obtain the same outcome whatever the chosen input.
- Two groups of dispersed inputs activate (black + green or black + blue), in these two scenarios the neuron fires.
- The two groups of clustered inputs (green + blue) activate, in this case the neuron remains silent as expected from our observation in Figure 4B.
- All inputs activate, in this last case the neuron firing rate remains moderate because of the refractory period.

This figure thus presents the response of the neuron model to all non-trivial cases, we have only omitted the case without any input activation (and therefore without any output activity).

**Figure 6.**
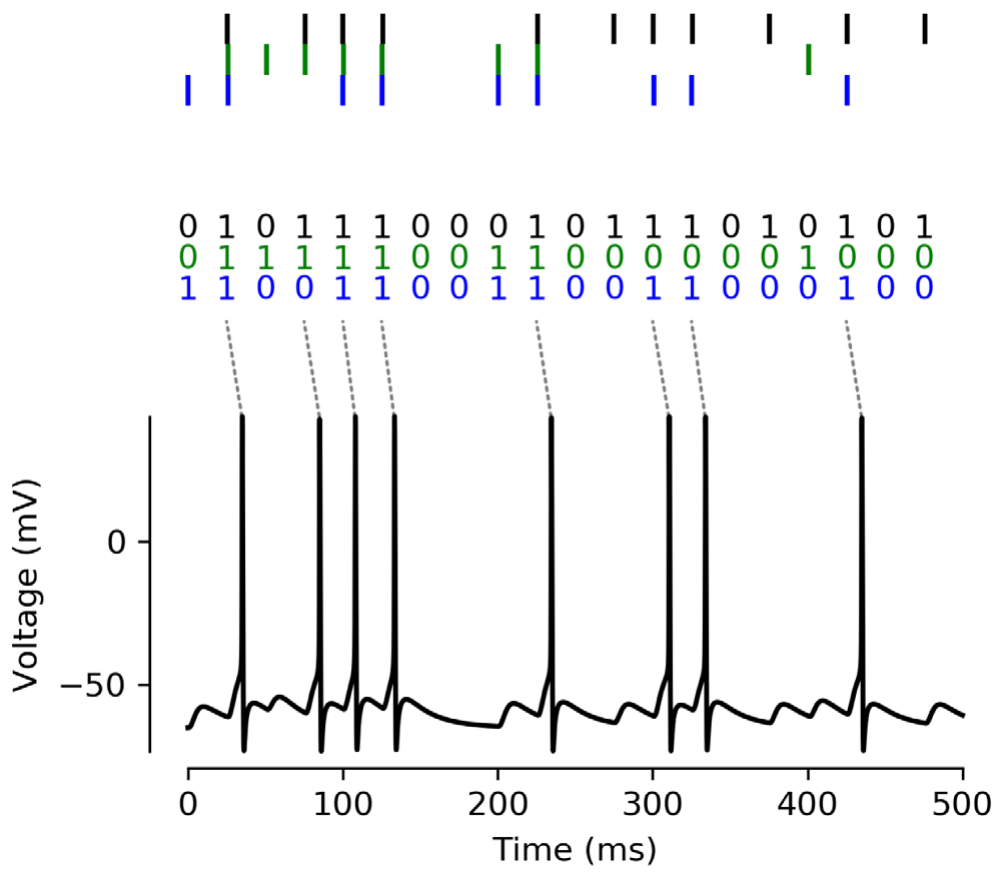
A biophysical model implementing the Dominant AND (spike interpretation). Top: The biophysical model receives input from three sources, where activation happens at regular intervals of 25 ms, with a random jitter of ±1ms for each spike. We translate this activity into a binary pattern for each time bin of 25 ms. Bottom: The model’s membrane potential as measured in the soma. The response spikes implement the output of the D-AND computation as described in Table 2.

Finally, we show an implementation of the spike interpretation in Figure 6. This model is identical to the model shown previously ( Figure 5), except for a slightly lower activation threshold of the sodium channels ( *V _T_* = −55 mV instead of *V _T_* = −50 mV) to make it spike more easily. We discretize time into bins of 25 ms and decide randomly for each input whether it is active in each bin. If it is active, it activates at the beginning of the bin with a small temporal jitter (1 ms); inputs activating in the same bin therefore spike coincidentally. We can directly link these activations to Boolean variables that are either 0 (no spike) or 1 (spike). As Figure 6 shows, the neuron implements the D-AND and only spikes whenever the black synapses activate together with at least one of the blue or green synapses.

We have shown that a biophysical model can implement the D-AND computation using a different strategy than the LTU. Each input has the same synaptic weight producing the same depolarisation at the soma. To distinguish between the inputs, the biophysical model uses location instead of strength: the dominant input (black) targets its own dendrite, while the two other inputs cluster on the same dendrite. With this strategy, the model can implement the D-AND. This implementation also works for two interpretations of the Boolean inputs and outputs – as elevated rates of spiking without temporal alignment, or as precisely timed coincident spikes. 

## Discussion

In the present work, we extend the linear threshold unit (LTU) to the sub-linear threshold unit (SLTU), a more realistic neuron model that includes non-linear processing in dendrites. We compare these two models on the implementation of a simple computation, the D-AND. We define it for three inputs and then extend it to *n* inputs by keeping its two defining features: a single dominant input that needs to be activated together with at least one of the remaining inputs. In this extension, the synaptic heterogeneity - e.g. the number of distinct binary synapses - grows linearly with *n* in the case of an LTU implementation while all synaptic weights remain equal for an SLTU with two dendrites.

For instance, if *n* = 1000 a single pre-synaptic input needs to make 999 synaptic contacts to implement the D-AND with a LTU while a single binary synapse suffices for a SLTU. This example demonstrates that a SLTU can implement the D-AND more efficiently -with less binary synapse - than the LTU.

Our denomination of one input as “dominant” and the others as “non-dominant” in the definition of the D-AND relates to the distinction between “driver” and “modulator” inputs ^20^. This concept, where driver inputs are necessary to activate a neuron, but this activity can be modulated by other inputs, is ubiquitous in the sensory system. For example, neurons in the primary visual cortex require a stimulus in their classical receptive field. Stimuli in the so-called extra-classical receptive field cannot activate the neuron by themselves, but strongly modulate the response if presented together with a stimulus in the classical receptive field ^21^. This distinction is not entirely applicable for the D-AND, since the dominant input *X*_1_ is not sufficient to activate the neuron by itself. Nevertheless, both computations rely on making a distinction between synaptic inputs, which can be implemented by placing inputs on different dendrites as we have shown in this study.

We show in a previous study that STLUs enable one to robustly implement a computation ^22^. In that study, an SLTU with eight dendrites implements direction selectivity while being resilient to massive synaptic failure. Alike the present work we exploited the placement of the synapses rather than the magnitude of their weight to implement the computation.

Several properties of our biophysical model used here fit with experimental observations. Firstly, synapses at different positions tend to create the same depolarisation at the soma ^18^. Secondly, while the depolarisation generated at a dendritic tip could be large (>50mV) the depolarisation recorded at the soma never exceeds 10mV. Finally, many experimental studies show examples of sub-linear summation in dendrites ^8, 9^, notably in interneurons.

How could neurons learn to implement the D-AND in an STLU? Multiple studies have shown that synaptic rewiring can happen at the sub-cellular level in a short time period ^23^ and that such a reorganisation could be used for learning ^24^. This markedly differs from classic Hebbian learning which uses changes in the total synaptic weight to implement computations.

Our biophysical model respects two important experimental observations. First, all synapses taken individually produce the same depolarisation at the soma, the so-called "synaptic democracy" like in 18. Second, several experimental studies show examples of sub-linear summationin dendrites ^10^, notably in interneurons ^8, 9^.

How could neurons learn to implement the D-AND in an SLTU? Multiple studies have shown that synaptic rewiring can happen at the sub-cellular level in a short time period ^23^ and that such a reorganisation could be used for learning ^24^. This markedly differs from classic Hebbian learning which uses changes in the total synaptic weight to implement computations, a SLTU friendly learning algorithm would keep the total synaptic weight constant while changing the targeted dendrites.

Our findings also have implications beyond neuroscience, in particular for engineering applications. Studies in computer science assert that even problems solvable by an LTU might not have a solution when weights have a limited precision ^25^. Being able to implement computations with an SLTU is therefore advantageous for hardware with limited resources.

In conclusion, dendrites unlock computations inaccessible without them and allow one to more efficiently implement the accessible ones. For instance, to implement the D-AND when n=1001 a SLTU needs a single synaptic contact for the dominant input while a LTU requires a thousand. Dendrites enable us to do more with less.

## Author contributions statement

R.C. wrote the initial draft, initiated the project, and made the initial simulations and figures. M.S. added additional simulations and improved part of the simulation code. Both authors discussed the results and wrote the final manuscript.
