# Supplementary material for: Neurons with dendrites can perform linearly separable computations with low resolution synaptic weights
Source: F1000Res. 2021 Apr 18;9:1174. Originally published 2020 Sep 28. [Version 3] doi: 10.12688/f1000research.26486.3 (PMC7848858; doi:10.12688/f1000research.26486.3)
Supplement: Peer review history

# Peer review: Reviewer response for version 3

**DOI:** 10.5256/f1000research.55803.r83470

**Published:** 2021-04-20

**Authors:** Fleur Zeldenrust

**Affiliations:** 1 Department of Neurophysiology, Donders Institute for Brain, Cognition and Behaviour, Radboud University, Nijmegen, The Netherlands

This paper shows, by fundamental derivation and simulations, that a certain class of neural computations can be more efficiently and realistically be implemented by neurons with dendrites than without dendrites. This is relevant and timely, as there has recently been an increased interest in dendritic computation, both from neuroscience and from neuromorphic computing. It is also a logical follow-up of the first author’s previous work, as mentioned in the discussion. I really appreciate the way of abstracting the neural computations to Boolean functions, as they also did in their previous work, which makes the work fundamental and generally applicable. The general setup of the paper is clear and appropriate, and the derivations and simulations are done correctly and clearly. My previous comments and concerns have all been addressed, and I now happily approve of this version.

Is the work clearly and accurately presented and does it cite the current literature?

Yes

If applicable, is the statistical analysis and its interpretation appropriate?

Not applicable

Are all the source data underlying the results available to ensure full reproducibility?

Yes

Is the study design appropriate and is the work technically sound?

Yes

Are the conclusions drawn adequately supported by the results?

Yes

Are sufficient details of methods and analysis provided to allow replication by others?

Yes

Reviewer Expertise:

Computational neuroscience

I confirm that I have read this submission and believe that I have an appropriate level of expertise to confirm that it is of an acceptable scientific standard.

# Peer review: Reviewer response for version 2

**DOI:** 10.5256/f1000research.50405.r77773

**Published:** 2021-03-10

**Authors:** Sacha van Albada

**Affiliations:** 1 Institute of Neuroscience and Medicine (INM-6), Research Centre Jülich, Germany; 2 Institute of Zoology, University of Cologne, Germany

I am happy with the changes and have no more comments on the content.

 I only still have a few tiny corrections:

 p. 3 “where most of the synapses lay” → “where most of the synapses lie”

 p. 6 “lays alone on a dendrite” → “lies alone on a dendrite”

 In Definition 5, the lower limits i=0 and j=0 should be i=1 and j=1.

 p. 8 “enable to robustly” → “enable one to robustly”

 p. 8, bottom: STLU → SLTU

 p. 9 “allow to more efficiently” → “allow one to more efficiently”

 Last sentence: “Dendrites enables” → “Dendrites enable”

Is the work clearly and accurately presented and does it cite the current literature?

Yes

If applicable, is the statistical analysis and its interpretation appropriate?

Not applicable

Are all the source data underlying the results available to ensure full reproducibility?

Yes

Is the study design appropriate and is the work technically sound?

Yes

Are the conclusions drawn adequately supported by the results?

Yes

Are sufficient details of methods and analysis provided to allow replication by others?

Yes

Reviewer Expertise:

computational neuroscience

I confirm that I have read this submission and believe that I have an appropriate level of expertise to confirm that it is of an acceptable scientific standard.

# Peer review: Reviewer response for version 2

**DOI:** 10.5256/f1000research.50405.r77774

**Published:** 2021-01-29

**Authors:** Fleur Zeldenrust

**Affiliations:** 1 Department of Neurophysiology, Donders Institute for Brain, Cognition and Behaviour, Radboud University, Nijmegen, The Netherlands

I appreciate the work the authors put in rewriting the text and reorganising the figures. I still think it is a nice and relevant story. I only have a few textual clarifications left.

**Figure 4:**

 I appreciate the reorganisation of the figure, and the addition of the ‘gray line’, but I think the caption and legend need to explain it a bit better. ‘Linear summation’ is not mentioned in the figure caption. More specifically:

 a/b) I see a white synapse, not a black one?

 b) I see only black-dashed and/or grey, not a black line? 

 “ In a linear neuron, all active groups of synapses (black + blue or black + green or blue + green) produce the same somatic EPSP (gray dotted line). “ — What does this mean? I see a clear difference between the aquamarine line and the black grey dashed line, but I don’t understand this sentence. Maybe you could specify how many synapses are activated?

 c) I don’t understand the difference between the first (black line — as far as I understand green+white or blue+white) and the second (green+white). Or the difference between the last (blue and green) and the penultimate (blue +green).  I also don’t see the gray dashed line in C. 

**Figure 5:**

 I appreciate the improved explanation of what was what, but I had to read this sentence a few times

 “(gray: no synapse/only black/green/blue, green: black + green, blue: black + blue, aquamarine: green + blue, black: all inputs active)”

 Also, as it is basically the same setup as in Fig 4 (right?), this might be mentioned. 

 “With linear summation these three input patterns would evoke identical responses.” -- I would add something like: ‘either all three would elicit not response (i.e. aquamarine line) or all three would electie a response (blue or green lines).

**Discussion:**

- In the present work, we oppose the linear threshold unit (LTU) to the sub-linear threshold unit (SLTU),— I don’t think you ‘oppose’ the LTU, you rather extend it.
- Remove ‘the’ before non-linear processing
- I appreciate the use of ‘heterogeneity’, but I think the term ‘synaptic heterogeneity’ needs to be defined — this term is used for the first time in the discussion as such, and needs a bit explanation here. Also, it needs to be explained here why the growth of the heterogeneity can be considered a problem (that needs to be solved by using for instance your SLTU). 
- “Our findings are in line with a previous study that demonstrated that SLTUs enable to robustly implement a computation 22. In that study, an SLTU with eight dendrites implements direction selectivity while being resilient to massive synaptic failure. As in the present work, findings were reproduced in a biophysical model.” Of the first sentence, the grammar is incorrect. Also, I don’t get the point. Now it reads as if the two studies are in line, because they were both reproduced in a biophysical model, but somehow I doubt that that was the point that you wanted to make. 
- ‘Several properties….fit with experimental observations’— could you please point to where these properties were used in your story? Especially the first: isn’t the main point of [18]  that this is the case despite the synapses being at different distances from the soma? Did you use that too? That wasn’t clear from the methods/results. The second point: now this is not any longer in figure 4, it is not clear how this relates to your model!
- Could you please explain how ‘synaptic rewiring’ could help neurons learn to implement for instance a D-AND, and how it differs from Hebbian learning? What do you mean exactly by rewiring, and when/how does this happen? I don’t need a whole review, but 2/3 sentences about how this could work would be nice. 
- Finally, you mention efficiency in both the introduction and the discussion. However, there is no efficiency calculation in your results. From my perspective, more efficiency means that you can do a computation with reduced cost. So which costs are reduced by using dendrites? Could you explain?

Is the work clearly and accurately presented and does it cite the current literature?

Yes

If applicable, is the statistical analysis and its interpretation appropriate?

Not applicable

Are all the source data underlying the results available to ensure full reproducibility?

Yes

Is the study design appropriate and is the work technically sound?

Yes

Are the conclusions drawn adequately supported by the results?

Yes

Are sufficient details of methods and analysis provided to allow replication by others?

Yes

Reviewer Expertise:

Computational neuroscience

I confirm that I have read this submission and believe that I have an appropriate level of expertise to confirm that it is of an acceptable scientific standard.

# Peer review: Reviewer response for version 2

**DOI:** 10.5256/f1000research.50405.r77772

**Published:** 2021-01-29

**Authors:** Panayiota Poirazi, Alexandra Tzilivaki

**Affiliations:** 1 Institute of Molecular Biology & Biotechnology, Foundation for Research & Technology - Hellas, Heraklion, Greece

The authors have successfully addressed all the previous comments and questions.

Is the work clearly and accurately presented and does it cite the current literature?

Yes

If applicable, is the statistical analysis and its interpretation appropriate?

Not applicable

Are all the source data underlying the results available to ensure full reproducibility?

Yes

Is the study design appropriate and is the work technically sound?

Yes

Are the conclusions drawn adequately supported by the results?

Yes

Are sufficient details of methods and analysis provided to allow replication by others?

Yes

Reviewer Expertise:

computational neuroscience, dendrites

We confirm that we have read this submission and believe that we have an appropriate level of expertise to confirm that it is of an acceptable scientific standard.

# Peer review: Reviewer response for version 1

**DOI:** 10.5256/f1000research.29243.r72169

**Published:** 2020-10-27

**Authors:** Panayiota Poirazi, Alexandra Tzilivaki

**Affiliations:** 1 Institute of Molecular Biology & Biotechnology, Foundation for Research & Technology - Hellas, Heraklion, Greece

In this modelling work, Caze and Stimberg propose that dendrites can efficiently decrease the weight resolution required to perform linear separable functions, something that is difficult to achieve using an LTU. The code that generates the model/data shown in figures is available and can be executed smoothly. All in all, the paper is a good fit for the F1000 Research. The results have the potential to advance the implementation of ANNs and neuromorphic hardware. Thus, it applies both to neuroscientific and computer science community.

 Suggestions:

1. The authors should test/report the robustness of their results by performing a sensitivity analysis for specific parameters of the model. For example, they could vary the number of dendrites, apply small changes in capacitance (Cm).
2. What would be effect of using negative synaptic weights in the LTU vs. the "dendritic neuron".
3. The term "dendritic neuron" is not ideal as it resembles dendritic cells, which are cells in the immune system. I would propose the use of a different term, e.g. "Neuronal dendrites" or "Neurons with dendrites".

Is the work clearly and accurately presented and does it cite the current literature?

Yes

If applicable, is the statistical analysis and its interpretation appropriate?

Not applicable

Are all the source data underlying the results available to ensure full reproducibility?

Yes

Is the study design appropriate and is the work technically sound?

Yes

Are the conclusions drawn adequately supported by the results?

Yes

Are sufficient details of methods and analysis provided to allow replication by others?

Yes

Reviewer Expertise:

computational neuroscience, dendrites

We confirm that we have read this submission and believe that we have an appropriate level of expertise to confirm that it is of an acceptable scientific standard, however we have significant reservations, as outlined above.

# Response

**Published:** 2021-01-20

**Authors:** Romain Cazé

**Affiliations:** CNRS UMR 8520, France

*The authors should test/report the robustness of their results by performing a sensitivity analysis for specific parameters of the model. For example, they could vary the number of dendrites, apply small changes in capacitance (Cm).*

 The results we present do not depend on model details, the only mandatory feature is the sub-linear summation in the dendrite. To further emphasize this point, we have added an exploration of the synaptic weight parameter in Fig.4 and discuss how it shows the robustness of the approach. Also note that the simulation code we provide can reproduce the basic results with a simple integrate-and-fire neuron model, further showing the robustness of the results.

     

*What would be effect of using negative synaptic weights in the LTU vs. the "dendritic neuron".*

 In general, introducing negative weights will require the weights of other synapses to be higher in order to compensate. Be aware that the D-AND function cannot be implemented (neither in the LTU nor in the SLTU) if one of the inputs only connects with a single synapse that has a negative weight. This is because for each input, there is at least one configuration of the other inputs where switching that specific input from 0 to 1 needs to switch the output result from 0 to 1. If it only connects with a negative weight, this is not possible.

    

*The term "dendritic neuron" is not ideal as it resembles dendritic cells, which are cells in the immune system. I would propose the use of a different term, e.g. "Neuronal dendrites" or "Neurons with dendrites".*

 We have changed the title to “Neurons with dendrites” to avoid any possible confusion.

# Peer review: Reviewer response for version 1

**DOI:** 10.5256/f1000research.29243.r72167

**Published:** 2020-10-23

**Authors:** Fleur Zeldenrust

**Affiliations:** 1 Department of Neurophysiology, Donders Institute for Brain, Cognition and Behaviour, Radboud University, Nijmegen, The Netherlands

This paper shows, by fundamental derivation and simulations, that a certain class of neural computations can be more efficiently and realistically be implemented by neurons with dendrites than without dendrites. This is relevant and timely, as there has recently been an increased interest in dendritic computation, both from neuroscience and from neuromorphic computing. It is also a logical follow-up of the first author’s previous work, as mentioned in the discussion. I really appreciate the way of abstracting the neural computations to Boolean functions, as they also did in their previous work, which makes the work fundamental and generally applicable. The general setup of the paper is clear and appropriate, and the derivations and simulations are done correctly and clearly. My main comments are minor and concern the presentation, concerning the language, some further explanations and a few citations. I will add them in detail below. Overall, if these minor edits are addressed I wholeheartedly recommend this paper.

 Detailed comments:

**P. 3**

- “a sufficiently large network of LTUs can approximate all possible computations” I think it depends a bit on the structure etc whether this is true (see https://en.wikipedia.org/wiki/Universal_approximation_theorem). Anyway, could there be a citation to how exactly this is meant?
- The formula you give for integer-valued weights does not give integer values? And n is not defined.
- The use of ‘means’ (in ‘finite means’) is a bit confusing: I think you mean something like ‘resources’, but you can also read it as ‘averages’.
- “Neuromorphic chips illustrate the problem and synapses often occupy the majority of the space, up to ten times more than the space occupied by neurons themselves” – I don’t really understand this sentence. How to these chips illustrate the problem? I also find the ‘and’ not logical, how does this belong together? I also miss a bit a conclusion, a ‘so…..’
- “Dendrites are the receptive elementS of neurons where most of the *receiving* synapses lay.”
- “They enable neurons to compute linearly inseparable computationS”
- “First, we investigate the three input variable computations implementable by an LTU”. I miss a sentence before this (something like: “We address this question by looking at…”). Also, what does ‘the three computations’ refer to? Which three computations? If there are only three, please include a reference or an explanation.
- “Third, we implement this computation at a smaller cost in..” Please define cost, otherwise it is meaningless.
- “This work not only shows the usefulness…” – better something like ‘a possible role’ or ‘a possible function’
- “and connect to the soma at their extremity” at ONE extremity.
- Merge paragraphs (i.e. delete white line) after eq. 2 (…compartments)
- Merge paragraphs (i.e. delete white line) after eq. 4 (… brevity)

**P. 4**

- “the code can also be run with a simpler leaky integrate-and-fire model.” Is that code included at the repository? If yes, say so explicitly, if not, delete this sentence.
- “peculiarities” replace with something like ‘the specific implementation’
- Definition 4: X ≥Z: what does this mean for vectors? The norm? Please specify.
- Definition 5: there are a few things unclear to me:
  - θ is defined but not used. Shouldn’t that be in the definition of E?
  - If the sum of w over j (wi) and all weights are either 0 or 1, then as soon as one synapse Xj with a weight wij is 1, E is 1, is that correct?
  - Y is not defined
  - I don’t see the sublinear summation here, can it be explained how this is sublinear summation? Also, a figure explaining this would really help, as it is one of the core definitions of the paper
- “computations that cannot be implemented in an LTU without using different strictly positive synaptic weights” ‘different’ is a bit confusing here (different from what?). Maybe ‘heterogeneous’?
- “We list all such computations in Table 2.” “Table 2. The five computations for n = 3 inputs”. I am a bit confused: do you mean all possible computations? How? I can fill in a random combination in that truth table, and then I have a computation you have not listed yet. So what do you mean? Could you explain this (or give a reference)?

**P. 5**

- “An LTU (Figure 1) implements the computation” which computation does this refer to? Because you describe DOR and DAND before
- “Here we always use” add comma between here and we
- Do not start a paragraph with ‘Then’. Also, ‘Next’ might be better
- “sub-linear summation can be observed in completely passive dendrites due to the intrinsic saturation of synaptic conductance” Isn’t this just an effect of the driving force, as also mentioned later in the results?
- “Here, the dominance of X0 is only expressed by its placement.” Please explain more
- Figure 2B: please refer to an equation or other explicit definition of the ‘saturating functions’

**P. 6**

- “…dendrites both integrate inputs sub-linearly in a given range. Therefore, we will focus on D-AND in the following section…” I don’t understand how the choice for D-AND follows from sublinear integration in dendrites.
- Figure 3B: please refer to an equation or other explicit definition of the ‘saturating functions’
- Figure 4
  - These EPSPs are huge! I understand why this is needed here (to get to the saturation), but it is not very biologically realistic. Could this be addressed in the discussion?
  - “…Membrane voltage traces responding…” a voltage trace does not respond.
  - “…locations (dendrite …” -> “ locations (at the dendrite…”
  - The locations of the arrows does not seem to quite correspond with the locations of measurements? At least the leftmost one should at the same position as the synapses (according to the text), not more towards the end of the dendrite, and I think the rightmost should be in the middle of the soma?
  - It would be nice if an example could be included to show that dendrites are needed, i.e. that it does not work without (saturating) dendrites
- Figure 5
  - Legend: part explaining the top should not be bold.
  - Top (activity of the inputs): for what input it this? 111?
  - It would be nice if an example could be included to show that dendrites are needed, i.e. that it does not work without (saturating) dendrites

**P. 7**

- Proof: there are two different fonts for Theta
- I’m not wild about the predator-prey analogy. I understand that you want a ‘real-world example’, but I think this one does not really apply, as something could not possibly be green and blue at the same time (and one would actually require some kind of mutual inhibition because of that). So I think this analogy only gives unnecessary confusion.
- Merge paragraphs (i.e. delete white line) ending with ‘…sublinear summation.’, ‘…at the soma.’ and ‘…response.’.
- Replace ; with ,  after individually.
- Add ‘-‘ with back propagation, or make one word.

P. 8

-  Merge paragraphs (i.e. delete white line) ending with ‘…spike interpretation.’
- Explanation Figure 5: so how is a synapse activated? Also not clear from figure.  Is it silent if it is not activated, and fires with 100 Hz if it is activated?
- Merge paragraphs (i.e. delete white line) ending with ‘…remain equal.’
- “The first two Results sections look at a computation and one of its possible extension”-> “In the first two sections in the Results we describe an example of a computation and one of its possible extentionS “ (a section does not look)
- “We think that the extension we chose is a reasonable one” Why? Please explain
- “Note also that…” do not start a paragraph that way.
- “Our study also relateS…”
- The discussion reads at the moment a bit more like a collection of loose arguments than as a single section. Could it be rewritten a bit so it is a bit more of a fluent story?
- Another discussion point that needs to be addressed in the discussion is plasticity and learning: whereas synaptic plasticity mechanisms that increase or decrease the synaptic weights are well known, plasticity mechanisms that change the locations of synapses much less, to my knowledge. So what would learning in such a system look like?

Is the work clearly and accurately presented and does it cite the current literature?

Yes

If applicable, is the statistical analysis and its interpretation appropriate?

Not applicable

Are all the source data underlying the results available to ensure full reproducibility?

Yes

Is the study design appropriate and is the work technically sound?

Yes

Are the conclusions drawn adequately supported by the results?

Yes

Are sufficient details of methods and analysis provided to allow replication by others?

Yes

Reviewer Expertise:

Computational neuroscience

I confirm that I have read this submission and believe that I have an appropriate level of expertise to confirm that it is of an acceptable scientific standard, however I have significant reservations, as outlined above.

# Response

**Published:** 2021-01-20

**Authors:** Romain Cazé

**Affiliations:** CNRS UMR 8520, France

We thank the reviewer for the thorough review and the constructive comments. We think we have addressed all the issues that have been raised, please find a detailed point-by-point reply below (original reviewer comments in *italics*):

*a sufficiently large network of LTUs can approximate all possible computations” I think it depends a bit on the structure etc whether this is true (see https://en.wikipedia.org/wiki/Universal_approximation_theorem). Anyway, could there be a citation to how exactly this is meant?*

 We decided not to go into details about the structure of such networks and therefore used "sufficiently large" to both encompass width and depth of the network. We now cite the study by G. Cybenko which (to our knowledge) is one of the first demonstrations of the finding.

*The formula you give for integer-valued weights does not give integer values? And n is not defined.*

 We have decided to no longer give the full formula, since its details are not relevant to our study (the formula could indeed give non-integer values, but it only established a lower bound). We now only state that the Hastad et al. “studied a computation implementable by an LTU only if its synaptic weight resolution grows exponentially with the number of inputs.”

*The use of ‘means’ (in ‘finite means’) is a bit confusing: I think you mean something like ‘resources’, but you can also read it as ‘averages’.*

 We have removed the consufing use of the word ‘means’ and replaced it by ‘resources’.

      

*Neuromorphic chips illustrate the problem and synapses often occupy the majority of the space, up to ten times more than the space occupied by neurons themselves” – I don’t really understand this sentence. How to these chips illustrate the problem? I also find the ‘and’ not logical, how does this belong together? I also miss a bit a conclusion, a ‘so…..’*

 We rewrote the paragraph to clarify our point (a large number of resources in LTU-based neuromorphic chips have to be dedicated to synapses).

    

*“First, we investigate the three input variable computations implementable by an LTU”. I miss a sentence before this (something like: “We address this question by looking at…”). Also, what does ‘the three computations’ refer to? Which three computations? If there are only three, please include a reference or an explanation.*

 We have rewritten the paragraph to make it clearer. The “three input variable computations” did not refer to three computations, but to computations of three input variables. We have rephrased this as “computations of three input variables” to avoid confusion.

      

*“Third, we implement this computation at a smaller cost in..” Please define cost, otherwise it is meaningless.*

 We have precise this point by stating that the implementation uses “fewer synapses”.

      

*“This work not only shows the usefulness…” – better something like ‘a possible role’ or ‘a possible function’*

 We have changed this sentence to state that the work “proposes a new role”.

      

*“and connect to the soma at their extremity” at ONE extremity.*

 We have change the sentence accordingly.

      

     Merge paragraphs (i.e. delete white line) after eq. 2 (…compartments)

      

     Merge paragraphs (i.e. delete white line) after eq. 4 (… brevity)

 We have merged the paragraphs and slightly changed the text surrounding the equations.

 P. 4

*“the code can also be run with a simpler leaky integrate-and-fire model.” Is that code included at the repository? If yes, say so explicitly, if not, delete this sentence.*

 Yes, The code in the repo can run both models. We have changed the text to make this point clearer.

* “peculiarities” replace with something like ‘the specific implementation’*

      

 We have changed the text accordingly

* Definition 4: X ≥Z: what does this mean for vectors? The norm? Please specify.*

     

 One vector is superior to another if all of its component are superior. This is specified as part of the definition (“meaning that for all i…”)

      

*Definition 5: there are a few things unclear to me:

θ is defined but not used. Shouldn’t that be in the definition of E?*

 Thank you for pointing this out. The θ was indeed meant to be the threshold in the function E, but for our purposes a fixed value of 1 is enough. We have therefore removed θ from the definition.

          

*If the sum of w over j (wi) and all weights are either 0 or 1, then as soon as one synapse Xj with a weight wij is 1, E is 1, is that correct?*

 This is correct and we now mention this in the text.

          

*Y is not defined*

 This was a printing error, Y is the argument of the function E (instead of X). We have corrected this.

          

* I don’t see the sublinear summation here, can it be explained how this is sublinear summation? Also, a figure explaining this would really help, as it is one of the core definitions of the paper*

 The summation is sublinear due to the saturation of the function E, which is the simplest (and strongest) sublinear summation: E(1) = 1, but E(1 + 1) = E(1 + 1 + …) = 1 as well. The shape of the function is now more clearly depicted in Figures 2 and 3.

* “computations that cannot be implemented in an LTU without using different strictly positive synaptic weights” ‘different’ is a bit confusing here (different from what?). Maybe ‘heterogeneous’?*

 We have rewritten this paragraph to make it clearer and now use ‘heterogeneous’ instead of ‘different’.

      

*“We list all such computations in Table 2.” “Table 2. The five computations for n = 3 inputs”. I am a bit confused: do you mean all possible computations? How? I can fill in a random combination in that truth table, and then I have a computation you have not listed yet. So what do you mean? Could you explain this (or give a reference)?*

 The table only lists positive threshold functions (or, equivalently, functions implementable by an LTU with non-negative weights), not all random combinations have this property. We have explained this more clearly in the rewritten paragraph.

 *P. 5*

*“An LTU (Figure 1) implements the computation” which computation does this refer to? Because you describe DOR and DAND before*

 Our statement refers to both computations, we have rewritten the sentence to make this explicit.

      

*“sub-linear summation can be observed in completely passive dendrites due to the intrinsic saturation of synaptic conductance” Isn’t this just an effect of the driving force, as also mentioned later in the results?*

 Yes, we have changed the sentence to refer to the driving force here as well.

      

*“Here, the dominance of X0 is only expressed by its placement.” Please explain more*

 We have expanded the explanation of the mechanisms used by the SLTU vs. the LTU.

      

*Figure 2B: please refer to an equation or other explicit definition of the ‘saturating functions’*

 We now refer to the definition of the function E in Def. 5.

 *P. 6

“…dendrites both integrate inputs sub-linearly in a given range. Therefore, we will focus on D-AND in the following section…” I don’t understand how the choice for D-AND follows from sublinear integration in dendrites.*

 We agree that this sentence was confusing and have removed it.

     

*Figure 3B: please refer to an equation or other explicit definition of the ‘saturating functions’*

 We now refer to Def. 5 in the caption.

      

 *Figure 4

These EPSPs are huge! I understand why this is needed here (to get to the saturation), but it is not very biologically realistic. Could this be addressed in the discussion?*

 These EPSPs were measured in the dendrite. When they are measured in the soma they are around 10mV and therefore more biologically realistic. While our reorganized Figure 4 no longer shows the EPSP measured at the dendrite, we now mention this point in the discussion.

          

*The locations of the arrows does not seem to quite correspond with the locations of measurements? At least the leftmost one should at the same position as the synapses (according to the text), not more towards the end of the dendrite, and I think the rightmost should be in the middle of the soma?*

 In our reorganized figure, the arrows are no longer needed.

          

*It would be nice if an example could be included to show that dendrites are needed, i.e. that it does not work without (saturating) dendrites*

 The reorganized Figure 4 now shows linear summation (i.e., without saturation) as a gray dotted line.

*Figure 5*

*Legend: part explaining the top should not be bold.*

**

*Top (activity of the inputs): for what input it this? 111?*

**

*It would be nice if an example could be included to show that dendrites are needed, i.e. that it does not work without (saturating) dendrites*

 We corrected the mistake in the caption and add a final sentence to explain what would happen in a point neuron: all the voltage responses (clustered or scattered) would  be equal.

 *P. 7*

*Proof: there are two different fonts for Theta*

 Thank you for alerting us to this issue, we will watch out for it in the revision proof.

*I’m not wild about the predator-prey analogy. I understand that you want a ‘real-world example’, but I think this one does not really apply, as something could not possibly be green and blue at the same time (and one would actually require some kind of mutual inhibition because of that). So I think this analogy only gives unnecessary confusion.*

 We agree and have removed the paragraph.

*P. 8*

*Explanation Figure 5: so how is a synapse activated? Also not clear from figure.  Is it silent if it is not activated, and fires with 100 Hz if it is activated?*

 Yes, an “active synapse” receives 25 spikes over 250ms (i.e. a 100Hz input) and “inactive synapses” do not receive any spikes. We now point this out in the Figure caption and changed the text to make it clearer.

      

*“We think that the extension we chose is a reasonable one” Why? Please explain*

 We have removed this sentence and instead now include a sentence describing the features of the function that are preserved by extending it in the way we did.

      

*The discussion reads at the moment a bit more like a collection of loose arguments than as a single section. Could it be rewritten a bit so it is a bit more of a fluent story?*

 We have rewritten parts of the Discussion to make it more fluent.

      

*Another discussion point that needs to be addressed in the discussion is plasticity and learning: whereas synaptic plasticity mechanisms that increase or decrease the synaptic weights are well known, plasticity mechanisms that change the locations of synapses much less, to my knowledge. So what would learning in such a system look like?*

 We have added a paragraph discussing the issue of plasticity to the discussion. We thank the reviewer for alerting us to a number of typos (missing ‘s’ at the end of words, missing commas, etc.) and language issues which we have corrected in the revised manuscript.

# Peer review: Reviewer response for version 1

**DOI:** 10.5256/f1000research.29243.r72166

**Published:** 2020-10-19

**Authors:** Sacha van Albada

**Affiliations:** 1 Institute of Neuroscience and Medicine (INM-6), Research Centre Jülich, Germany; 2 Institute of Zoology, University of Cologne, Germany

This paper shows that having synaptic inputs onto separate dendrites can lower the resolution and spread of the synaptic weights necessary for performing computations. The authors illustrate this using both simple threshold units and biophysical neuron models, on the example of a particular function they call ‘dominant AND’. The work appears to be novel and correct, and is presented in a well-organized, comprehensible manner. It may have an impact not only in neuroscience but also on the implementation of artificial neural networks.

**Minor**

 The condition on the weights given in the second paragraph of the introduction was only derived under certain conditions (n>=8 a power of 2) and it is still only a minimal lower bound – some threshold functions might still require larger weights. It should thus not be presented as a general condition.

 The work of Ujfalussy *et al. *seems relevant but is not cited.

 The English can be improved in places. For instance in the 2 ^nd^ paragraph of the Introduction: “synaptic weights resolution” → “synaptic weight resolution”, “compute all ...computation” → “perform all...computations”, “they evolve...with” → “the weights depend...on”. Also in other places, reword “compute computations”.

 In the same paragraph “an LTU needs integer-valued weights” is a bit misleading. Of course the weights do not need to be integer-valued, they can just be taken to be integer-valued without loss of generality. In the last sentence of that paragraph, please clarify what you mean by “means”.

 “The dynamics of the gating variables...are adapted from...and omitted here for brevity”: Please clarify if the dynamics are taken to be identical to the dynamics in that reference (“adapted” suggests otherwise – in which case more details would be needed for completeness).

 In Definition 2, I would write “at least one vector” instead of “at least a vector”.

 In Definition 5, maybe instead of “n inputs” you want to write “n inputs onto each dendrite”, and in the sum over w_{i,j} indicate the limits of j (presumably 1 to n). “d dendritic threshold” → “d dendritic thresholds”. In the first equation of Def. 5, you use limits 0 and d, and 0 and n, corresponding to (d+1) * (n+1) weights, instead of d * n, so I suggest adjusting the limits. In the second equation of Def. 5, you use X on the left-hand side but Y on the right-hand side.

 “provided a sufficient number of dendrites and synapses” → “given a sufficient number of dendrites and synapses”

 “It allows to...” → “This allows one to...” or “This allows us to...”

 In the same sentence, please clarify what you mean by “similar synapses”.

 Fig. 1 caption “compare to the others” → “compared to the others”

 “the other three computations which treat” → “the other three computations, which treat”

 p. 6, top: The link between focusing on D-AND and the focus on sublinearity is unclear.

 Fig. 4 caption: ‘coloured squares depicts’ → ‘coloured squares depict’

 p. 7, top: ‘greater or equal than’ → ‘greater than or equal to’

 p. 7: ‘If one would only measure’ → ‘If one only measured’

 p. 8: ‘it has now active’ → ‘it now has active’

 Please be clearer on the role of the active sodium channels in supporting the computation. The abstract speaks of passive dendrites.

 I would reword ‘the neuron does not overly fire notably’ to something like ‘the output firing rate remains moderate’.

 Discussion: ‘many times higher than the other’ → ‘many times higher than the lowest of the other weights’ or similar

 ‘one of its possible extension’ → ‘one of its possible extensions’

 The sentence ‘In conclusion, dendrites...than without’ is not grammatically correct.

Is the work clearly and accurately presented and does it cite the current literature?

Yes

If applicable, is the statistical analysis and its interpretation appropriate?

Not applicable

Are all the source data underlying the results available to ensure full reproducibility?

Yes

Is the study design appropriate and is the work technically sound?

Yes

Are the conclusions drawn adequately supported by the results?

Yes

Are sufficient details of methods and analysis provided to allow replication by others?

Yes

Reviewer Expertise:

computational neuroscience

I confirm that I have read this submission and believe that I have an appropriate level of expertise to confirm that it is of an acceptable scientific standard, however I have significant reservations, as outlined above.

# Response

**Published:** 2021-01-20

**Authors:** Romain Cazé

**Affiliations:** CNRS UMR 8520, France

*The condition on the weights given in the second paragraph of the introduction was only derived under certain conditions (n>=8 a power of 2) and it is still only a minimal lower bound – some threshold functions might still require larger weights. It should thus not be presented as a general condition.*

 We rewrote this part of the introduction in less technical term while improving its accuracy.

*The work of Ujfalussy et al. seems relevant but is not cited.*

 We now cite a work their work in the introduction

*The dynamics of the gating variables...are adapted from...and omitted here for brevity”: Please clarify if the dynamics are taken to be identical to the dynamics in that reference (“adapted” suggests otherwise – in which case more details would be needed for completeness)*.

 We changed adapted to “identical, except for…”, detailing the one difference to the parameters used in the reference.

In Definition 2, I would write “at least one vector” instead of “at least a vector”. In Definition 5, maybe instead of “n inputs” you want to write “n inputs onto each dendrite”, and in the sum over w_{i,j} indicate the limits of j (presumably 1 to n). “d dendritic threshold” → “d dendritic thresholds”. In the first equation of Def. 5, you use limits 0 and d, and 0 and n, corresponding to (d+1) * (n+1) weights, instead of d * n, so I suggest adjusting the limits.

 We implemented the changes and corrected our definition and kept these changes throughout the article.

*In the second equation of Def. 5, you use X on the left-hand side but Y on the right-hand side.*

 These mistakes happened during the editorial process, we corrected them.

*Pp. 6, top: The link between focusing on D-AND and the focus on sublinearity is unclear.*

 We upgraded figure two and three, and changed the text to make this link clearer.

 Finally, we corrected all the English mistakes underlined by the reviewer, clarified it, reworded the article to avoid compute/computation and we clarified what we meant by "means".
